# Supplementary material for: MetaRibo-Seq measures translation in microbiomes
Source: Nat Commun. 2020 Jun 29;11:3268. doi: 10.1038/s41467-020-17081-z (PMC7324362; doi:10.1038/s41467-020-17081-z)
Supplement: Supplementary file 10 — Supplementary Data 7 [file 41467_2020_17081_MOESM10_ESM.zip › File2/Confidence_High_Taxonomy/379256_out.krona.html]

Javascript must be enabled to view this page.

members
magnitude
magnitudeUnassigned
count
unassigned
taxon
rank

379256\_out

3

2
superkingdom
3

3
phylum
1239

186801
class
3

3
186802
order

2

SRS018427\_contig\_number\_23152SRS147346\_contig\_number\_63597
1715004
species

1
1897043
species

SRS015431\_contig\_number\_61986
